# Supplementary material for: Neurological Damage Measured by S-100b and Neuron-Specific Enolase in Patients Treated with Electroconvulsive Therapy
Source: Brain Sci. 2024 Aug 16;14(8):822. doi: 10.3390/brainsci14080822 (PMC11352697; doi:10.3390/brainsci14080822)
Supplement: Supplementary file 1 [file brainsci-14-00822-s001.zip › brainsci-3056377-supplementary.pdf]

Supplementary material.

Characteristics of Electrical Stimulus Generation

Waveform: Brief biphasic square wave pulse

Current: 0.9 constant amps, for over 500 ohms of impedance

Voltage: Proportional to dynamic impedance, max. 450 V

Frequency: Standard: 70 Hz at 55-100% power

50 Hz at 30-50% power

30 Hz at 5-25% power

FlexiDial: 30, 40, 50, 60, 70 Hz

Pulse Amplitude: Standard: 1.0 msec

With FlexiDial: 0.5, 1.0, 1.5 msec

Duration: Standard 0.47 to 4 sec

With FlexiDial: 0.1 to 8 sec.

Energy: 220 ohm impedance: 4.97 J to 99.4 J

Charge: 25.2 to 504 milliCoulombs in 20 equal steps on the % energy dial.

Impedance Test: 0 to 3000 ohms (+/- 100 ohms) at 1000 Hz

Seizure Monitoring.

Channel Specifications (for each channel):

Sensitivity: Gain = 80 dB

Frequency Response: 2 to 50 Hz, 60 Hz with node filter

Common-mode Rejection: 80 dB

Auxiliary Output: +/- 10 v, full scale

Complete isolation for opto electronics

Channel selection: Channel 1: EEG Channel 2: EEG, EKG, EMG.

Thermal paper graduation speed 25 mm/sec

Convulsive Quality Measures:

Seizure Energy Index (EEG: integrated voltage of broadband ictal EEG)

Postictal Suppression Index (EEG: from 0 to 100)

Convulsive Concordance Index: (EEG: from 0 to 100)

The equipment is located in a dedicated ECT room equipped with: Cardiopulmonary resuscitation equipment, suction equipment, oxygen outlets, cardiac monitoring and defibrillator equipment, as well as pulse oximeter, heart rate, and blood pressure monitoring.

Application Technique of ECT at INNN consists of:

1. Obtaining signed informed consent from the patient's legal guardian, a witness, and the treating physician.

2. The previously described clinimetry was conducted both before and after ECT to monitor cognitive side effects.

3. Anesthetic assessment with blood tests, QS, ES, EGO, EKG, TP, and TPT.

4. CT scan if patients do not have one.

5. EEG if patients do not have one.

6. Electrocardiogram and chest X-ray for patients over 45 years old.

7. Reduction of medication that pharmacologically interacts with ECT.

8. Fasting from 22:00 (day before ECT)

9. Morning bath with strict hair drying.

10. Cannulation with 0.9% saline solution

11. Pre-treatment Blood Pressure measurement

12. Sedation with Thiopental calculated at 5 mg/kg/dose

13. Placement of tourniquet on upper limb where electrodes will be placed for electro-myographic (EMG) activity measurement.

14. Muscle relaxation with succinylcholine

15. Skin preparation with acetone for placement of bitemporal electrodes for electrical stimulation discharge.

16. Placement of channel 1 electrodes for Electroencephalographic monitoring.

17. Placement of electrodes over the common flexor muscle of the fingers with an approximate distance of 15 cm for EMG.

18. Placement of ground electrode in the region of the sternal manubrium.

19. Following fasciculations after succinylcholine administration, impedance is verified.

20. The equipment is designed not to deliver electrical discharge above 3000 ohms of impedance.

21. The energy level is selected, considering the protocol that determines the necessary percentage to initiate the first ECT session (as mentioned earlier, based on the patient's age).

22. A mouth guard is placed, and 100% oxygen is administered.

23. The discharge is delivered, with visual (via EEG recording) and auditory verification of the seizure.

24. Blood pressure, heart rate, and respiratory rate are measured during the seizure. The neuroanesthesiologist provides ventilation.

25. A seizure lasting more than 25 seconds is considered effective. If the duration exceeds 120 seconds, it is terminated with Diazepam.

26. If a seizure lasting more than 25 seconds is not achieved, immediate re-stimulation occurs, if there are no contraindications from the anesthesiologist, considering an effective cumulative effect with the sum of two seizures lasting over 25 seconds.

27. If the patient's blood pressure rises above 150/110 after 5 minutes of seizure termination, 10 mg IV esmolol is administered.

28. The seizure is considered terminated (electroencephalographic activity) when the trace is flattened, and there is an absence of audible signal.

29. The patient is kept under recovery and surveillance by the neuroanesthesiologist and attending nurse until adequate secretion management and ventilatory automatism are observed.

30. Finally, the patient is transferred to their bed and remains there until full recovery.

Choice of Electrode Placement Modality for Electroconvulsive Therapy

Although bilateral placement, primarily in elderly patients, is known to have cognitive complications (recent memory loss, confusion episodes), we use this technique in the department due to supply issues with consumables.

Registering of Electroconvulsive Therapy in the Database

The stimulus delivered, electroencephalographic activities of the indicated sessions for patients, and the number of ECT sessions were registered in the database.
